# KCC2 inhibition and neuronal hyperexcitability promote extrinsic apoptosis dependent upon C1q

**DOI:** 10.3389/fnmol.2025.1645428

**Published:** 2025-08-18

**Authors:** Jinglin Ji, Catherine Choi, Christopher E. Bope, Jacob S. Dengler, Stephen J. Moss, Joshua L. Smalley

**Affiliations:** Department of Neuroscience, Tufts University School of Medicine, Boston, MA, United States

**Keywords:** neuroimmunology, epilepsy, apoptosis, potassium chloride co-transporter 2, complement C1q

## Abstract

**Introduction:**

The potassium chloride co-transporter 2 (KCC2) is the principal Cl^−^ extrusion mechanism employed by mature neurons in the central nervous system (CNS) and plays a critical role in determining the efficacy of fast synaptic inhibition mediated by type A *γ*-aminobutyric acid receptors (GABA_A_Rs) to protect against epileptogenesis. It has previously been demonstrated that epileptic seizures down-regulate KCC2 and induce neuronal apoptosis through the extrinsic apoptotic pathway. However, the mechanism by which neuronal death is induced by KCC2 loss remains unknown. We have previously demonstrated that C1q copurifies with KCC2 in comparable amounts. C1q is responsible for synaptic elimination in the brain during development, aging and neurodegeneration.

**Methods:**

Here, we studied apoptotic induction in models of KCC2 loss of function and demonstrated the importance of C1q in this process using a constitutive C1qKO mouse model. We characterized the activation of different apoptotic pathways by measuring caspase 8 and caspase 9 cleavage as markers of extrinsic and intrinsic apoptosis, respectively.

**Results:**

This study demonstrates *in vitro, ex vivo* and following seizures *in vivo*, that reduced KCC2 function coincides with neuronal death by activating the extrinsic apoptotic pathway, which is contingent upon complement C1q. Moreover, kainic acid (KA)- and glutamate-induced excitotoxicity also selectively activates the extrinsic apoptotic pathway which is contingent upon C1q.

**Discussion:**

These results strongly support the hypothesis that the KCC2/C1q protein complex plays a critical role in the apoptotic process that occurs following loss of KCC2 function.

## Introduction

Fifty million people worldwide are affected by epilepsy, which involves the imbalance of neuronal excitation and inhibition (World Health Organization, 2006; Shao et al., 2019). Increased excitation, decreased inhibition, or both favor a hyperexcitable state and an increased propensity for seizure generation and epileptogenesis (World Health Organization, 2006; Shao et al., 2019). Type A *γ*-aminobutyric acid receptors (GABA_A_Rs) represent the major inhibitory receptors in the nervous system, and the inhibitory effect of GABA_A_Rs plays an important role in the maintenance of normal brain function and protection against epileptogenesis. As fast-acting ligand-gated ion (Cl^−^) channels, GABA_A_Rs mediate fast synaptic inhibition via chloride ion influx, and the subsequent hyperpolarization of neurons. This influx of chloride ions relies on low intracellular Cl^−^ and high extracellular Cl^−^ transmembrane gradient, which is established through Cl^−^ extrusion by potassium chloride co-transporter 2 (KCC2) in mature neurons (Chamma et al., 2012; Mahadevan and Woodin, 2016; Wu et al., 2016). KCC2, encoded by the gene *SLC12A5*, is the principal Cl^−^-extrusion mechanism in the central nervous system (CNS) that enable GABA_A_R-mediated inhibition (Moore et al., 2017). Human patients with mutations in the *SLC12A5* gene develop severe epilepsy soon after birth and developmental delay, while deletion of the *SLC12A5* gene in mice causes hyperexcitability in the hippocampus, generalized seizures, temporal lobe epilepsy, and death shortly after birth (Kelley et al., 2018; Chen et al., 2017; Uvarov et al., 2007; Woo et al., 2002; Hubner et al., 2001; Stodberg et al., 2015; Saitsu et al., 2016; Saito et al., 2017). In short, KCC2 plays a critical role in determining the efficacy of fast synaptic inhibition mediated by GABA_A_Rs and protect against epileptogenesis (Moore et al., 2017).

Apoptosis is a coordinated form of cell death that occurs during development or normal tissue homeostasis (Kruger and Richter, 2022). There are two major signaling pathways that lead to apoptosis: the intrinsic and extrinsic pathways. The extrinsic pathway is triggered by extracellular ligands through cognate death receptors at the surface of target cells, while the intrinsic pathway is usually activated by an injury occurring within the cell (Nair et al., 2014). In both the extrinsic and intrinsic pathways of apoptosis, signaling activates a family of cysteine proteases; caspases, that act via a proteolytic cascade to dismantle and remove the dying cells (Peter, 2011). While the final stages of the apoptotic cascade are similar between intrinsic and extrinsic apoptosis, such as the cleavage of caspases 3, caspase 7, and poly (ADP-ribose) polymerase (PARP), the upstream initiator caspases and protein complexes differ (Slee et al., 2001). For instance, caspase 8 is activated in response to death receptor activation, serving as a marker of extrinsic apoptosis, while caspase 9 is specific for the intrinsic pathway (Cohen, 1997).

Previous work has demonstrated that KCC2 is required for the survival of mature neurons and is crucial in the regulation of seizure-induced neuronal death (Kelley et al., 2018; Kontou et al., 2021). It has previously been shown that epileptic seizures down-regulate KCC2 and induce neuronal apoptosis through the extrinsic pathway (Teocchi and D'Souza-Li, 2016; Meller et al., 2006; Gonzalez, 2016; Henshall, 2007). The inactivation of KCC2 in the hippocampus *in vivo* and in mature neurons *in vitro* induces neuronal loss and activates the extrinsic apoptotic pathway (Kelley et al., 2018; Kontou et al., 2021). However, the underlying mechanism by which neuronal death is induced by KCC2 expression ablation or function loss remains unknown (Henshall, 2007).

To investigate the mechanism that links KCC2 activity to neuronal apoptosis, the major interactors of KCC2 were screened using quantitative proteomics (Smalley et al., 2020). Notably, comparable amounts of complement C1q and three main subnetworks containing complement C1q were detected in high molecular weight stable multi-protein complexes of KCC2 at a ratio of 1:1.58 KCC2: C1q (Smalley et al., 2020). This observation indicated the fascinating possibility that KCC2 is involved in neuro-immune interactions, especially as KCC2 has been shown to be near excitatory and inhibitory synapses, and C1q is known to be involved in synaptic elimination. C1q is formed from 3 subunits of 6 peptide chains, with each subunit consists of a Y-shaped pair of triple peptide helices (C1qA, C1qB, and C1qC chains) joined at the stem and ending in a globular non-helical head. C1q is the initiating protein of the classical complement cascade, a powerful effector of the innate immune system responsible for pathogen targeting and removal. C1q has also been shown to induce apoptosis in tumor-derived cell lines (Hong et al., 2009; Kaur et al., 2016; Ricklin et al., 2010). In the CNS, C1q is differentially expressed in neurons, astrocytes, oligodendrocytes, and microglia (Tenner et al., 2018; Schartz and Tenner, 2020). The complement cascade, initiated by C1q, is responsible for selective CNS synapse elimination during development, aging, and neurodegeneration. C1q induced synapse elimination occurs via the deposition of activated downstream complement protein C3, also known as synapse opsonization, leading to either direct cell lysis or microglia-mediated phagocytosis of the opsonized synapses via engagement of synapse-bound iC3b and the microglial CR3 complement receptor (CD11b/CD18) (Bruce-Keller, 1999; Bhakdi and Tranumjensen, 1987; Stevens et al., 2007; Schafer et al., 2012).

Given the essential role of C1q in mediating synaptic elimination and neuronal loss in the brain, insight into the role that C1q plays in regulating KCC2 function and neuronal viability is critical to characterize the mechanism of neuronal death following that occurs following loss of KCC2 function. Here, we studied apoptotic induction in models of KCC2 loss of function and demonstrated the importance of C1q in this process using a constitutive C1qKO mouse model. We characterized the activation of different apoptotic pathways by measuring caspase 8 and caspase 9 cleavage as markers of extrinsic and intrinsic apoptosis, respectively. Neuronal death induced following seizures is a major deleterious event that results in long-term compromise of patients’ life quality. Therefore, by characterizing the role of the KCC2/C1q axis in regulating neuronal viability and apoptotic induction following seizures will help to derive novel therapeutic strategies that prevent neuronal loss. Therapeutic KCC2 activators are also on the horizon, and this study will help inform important pre-clinical endpoints that can be used to establish their efficacy.

## Results

### Acute inhibition of KCC2 by the specific KCC2 inhibitor 11 K induces neuronal death

11 K is a potent (KCC2 IC_50_ = 61 nM) and selective (>100-fold versus NKCC1) KCC2 inhibitor that inhibits KCC2 activity and induces rapid neuronal depolarization, resulting in epileptiform discharges (Agez et al., 2017; Pegurier et al., 2010; Moore et al., 2018; Kelley et al., 2016; Sivakumaran et al., 2015). The inhibition of KCC2 activity by 11 K in mature neurons which already exhibit KCC2-dependent hyperpolarizing GABA_A_R currents, mimics the acute loss of KCC2 following seizures (Moore et al., 2017; Kelley et al., 2018; Jaenisch et al., 2010; Huberfeld et al., 2007). Previous *in vitro* studies have shown that acute inhibition of KCC2 in mature neurons by 11 K raises the levels of the early extrinsic apoptotic marker cleaved caspase 8 within 10 min and activates the extrinsic apoptotic pathway (Kontou et al., 2021). Here, for the first time we verified *in vitro* the neuronal loss induced by acute KCC2 inhibition in mature neurons using TUNEL staining, and demonstrated the specificity of the KCC2 inhibitor 11 K.

**Figure 1:**
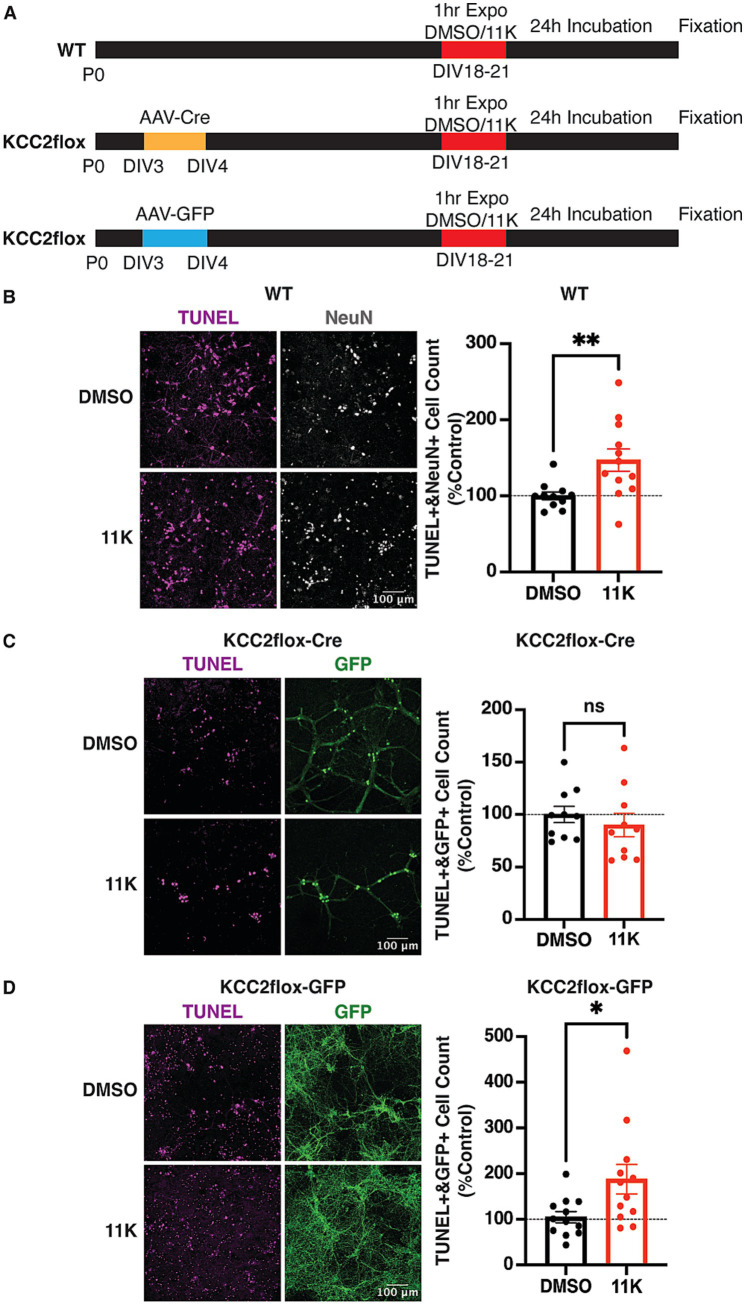
Verification of the neuronal loss induced by KCC2 inactivation and the specificity of the KCC2 inhibitor 11 K. **(A)** schematic diagram of the AAV-virus transduction and treatment timeline. **(B)** TUNEL and ICC staining and quantification of TUNEL+ and NeuN+ WT neurons exposed to 11 K (1 μM) or DMSO (0.1%) for 1 h at DIV18-21. **(C)** TUNEL and ICC quantification of TUNEL+ and GFP + KCC2^flox^ neurons infected with AAV-Cre-GFP at DIV3. **(D)** TUNEL and ICC quantification of TUNEL+ and GFP + KCC2^flox^ neurons infected with AAV-GFP at DIV3. The results were expressed as mean ± the SEM. All data were subjected to the F-test to compare variances on GraphPad Prism (Version 9.2.0). To assess statistical significance, the unpaired two-sample t-tests were performed, and the Welch’s corrections were applied when necessary. Significance of *p* < 0.05 was represented as *, *p* < 0.01 was represented as **, *p* < 0.001 was represented as ***, and *p* < 0.0001 was represented as ****. All replicates were independent biological replicates. n = 4 individual primary cultures from 6 to 12 pups per genotype, scale bar = 100 μm.

To examine the effect of acute inhibition of KCC2 on neuronal viability, days in vitro (DIV) 18–21 mixed hippocampal/cortical cultured WT neurons were exposed to 11 K (1 μM) or DMSO (0.1%) for 1 h, and incubated in new conditioned media for 24 h prior to fixation (Figure 1A). Apoptotic neurons were quantified by TUNEL staining and normalized to the DMSO treated control group from the same primary culture preparation. The neuronal nuclear protein NeuN was used as a general cell marker (Verdiev et al., 2009; Petrova et al., 2014; Petrova and Isaeva, 2014; Korzhevskii et al., 2009; Gusel’nikova and Korzhevskiy, 2015). TUNEL+ and NeuN+ WT neurons were significantly higher in the 11 K treated group compared to the DMSO control, verifying that acute inhibition of KCC2 induces neuronal death (Figure 1B).

To test the specificity of 11 K inhibition on KCC2, KCC2^flox^ neuronal cultures were used as a control, in which KCC2 expression was ablated via Cre-recombinase-mediated excision of exons 22–25 of the *SLC12A5* gene as previously described (Mercado et al., 2006; Acton et al., 2012). This truncation of the *SLC12A5* gene has been verified to cause complete ablation of KCC2 expression (Kelley et al., 2018; Kontou et al., 2021). KCC2 expression in rodent brains is developmentally regulated with low levels before birth and then dramatically increasing from P7 onwards (Blaesse et al., 2009). Previous studies have shown that removing KCC2 in immature neurons at DIV3 does not affect neuronal viability, dendritic arborization, or spine formation (Kontou et al., 2021). Therefore, DIV18–21 KCC2^flox^ neuronal cultures infected with AAV-Cre at DIV3 served as a control to demonstrate the specificity of 11 K for KCC2 (Figure 1A).

AAV-Cre infected KCC2^flox^ cultures showed no significant difference in TUNEL cell counts between DMSO and 11 K treatment groups (Figure 1C). The cell density of primary neurons cultured in the absence of KCC2 is generally lower, due to the importance of KCC2 in establishing neuronal networks. 11 K exposure induced cell death only in WT but not in KCC2 KO mature neurons, verifying the specificity of 11 K for KCC2. In comparison, TUNEL+ cell counts were significantly increased by 11 K in AAV-GFP infected cultures, like the effect of 11 K on WT cultures (Figure 1D). Therefore, the increase in neuronal loss after 11 K exposure is dependent on KCC2.

### Acute inhibition of KCC2 selectively activates the extrinsic apoptotic pathway, without affecting the intrinsic apoptotic pathway

**Figure 2:**
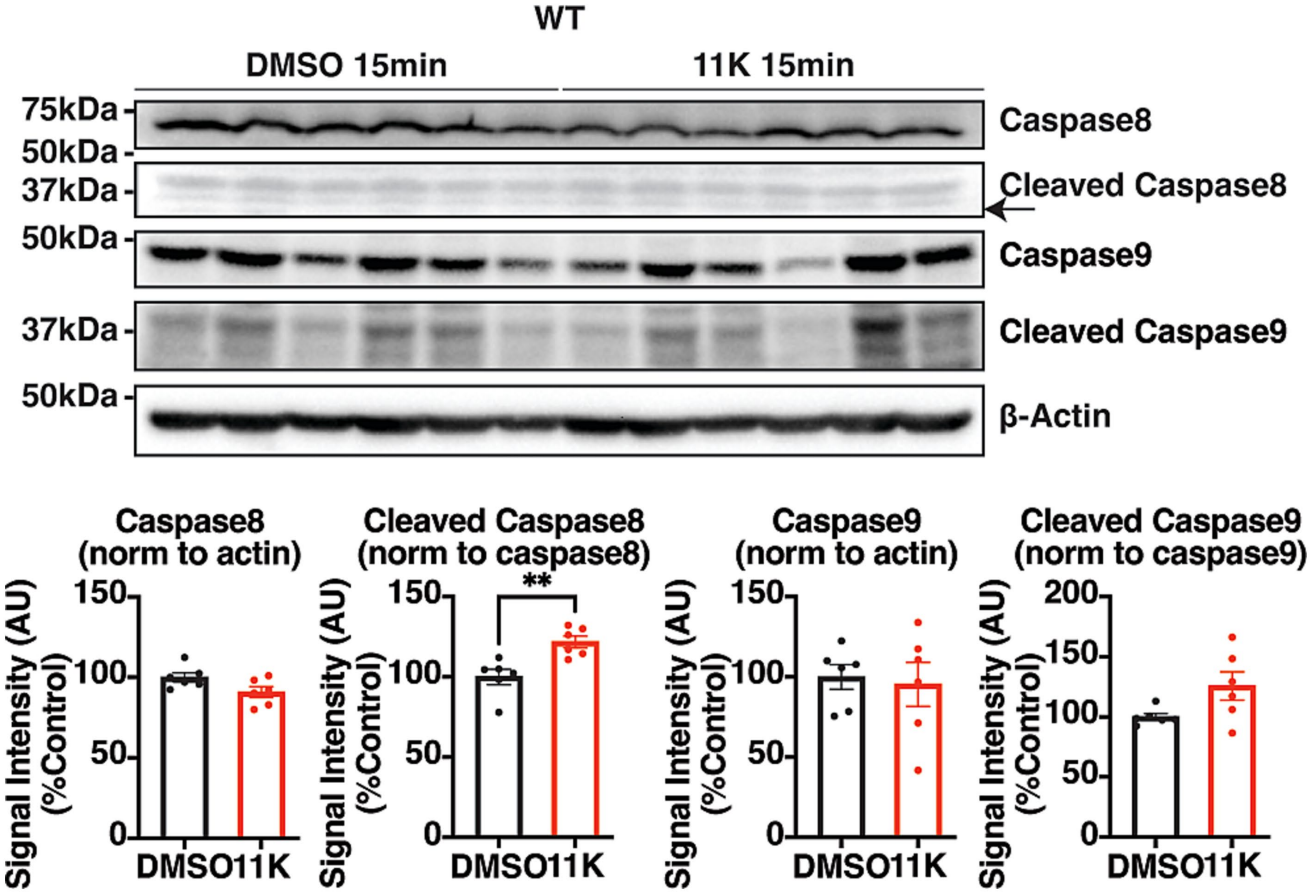
Identification of the apoptotic pathway activated by the acute inhibition of KCC2. Representative IB images and quantifications of pro- and cleaved caspase 8 and caspase 9 in 350 μm coronal hippocampal acute slices from adult WT mice. Brain slices were exposed to 11 K (10 μM) or DMSO (0.1%) for 15 min, and lysed with 100 μL RIPA lysis buffer per slice. The results were expressed as mean ± the SEM. All data were subjected to the F-test to compare variances on GraphPad Prism (Version 9.2.0). To assess statistical significance, the unpaired two-sample t-tests were performed, and the Welch’s corrections were applied when necessary. Significance of *p* < 0.05 was represented as *, *p* < 0.01 was represented as **, *p* < 0.001 was represented as ***, and *p* < 0.0001 was represented as ****. All replicates were independent biological replicates. *n* = 6 animals per treatment group, 2–3 slices (merged) per animal.

To identify the activation of different apoptotic cascades in a physiologically intact system where neuronal networks are preserved, hippocampal acute brain slices from adult WT mice were treated with 11 K for 15 min. The cleavage of caspase 8 and caspase 9 was quantified using IB and compared to the DMSO control group. Cleavage of the early extrinsic apoptotic marker caspase 8 generates p43/41 cleaved caspase 8, initiating the extrinsic apoptotic pathway (Kallenberger et al., 2014). Acute inhibition of KCC2 by 11 K marginally reduced the levels of procaspase 8 and significantly raised the levels of cleaved (p41) caspase 8 within 15 min, both of which indicate activation of the extrinsic apoptotic pathway. No significant change was observed in the levels of procaspase 9 nor the cleavage of caspase 9, which is specific for the activation of the intrinsic apoptotic pathway (Figure 2). Therefore, the acute inhibition of KCC2 selectively activates the extrinsic apoptotic pathway without affecting the intrinsic apoptotic pathway in acute brain slices. This result is consistent with previous findings and that demonstrate this in cultured neurons.

### The activation of the extrinsic apoptotic pathway induced by acute KCC2 inhibition is contingent upon neuronal activity

**Figure 3:**
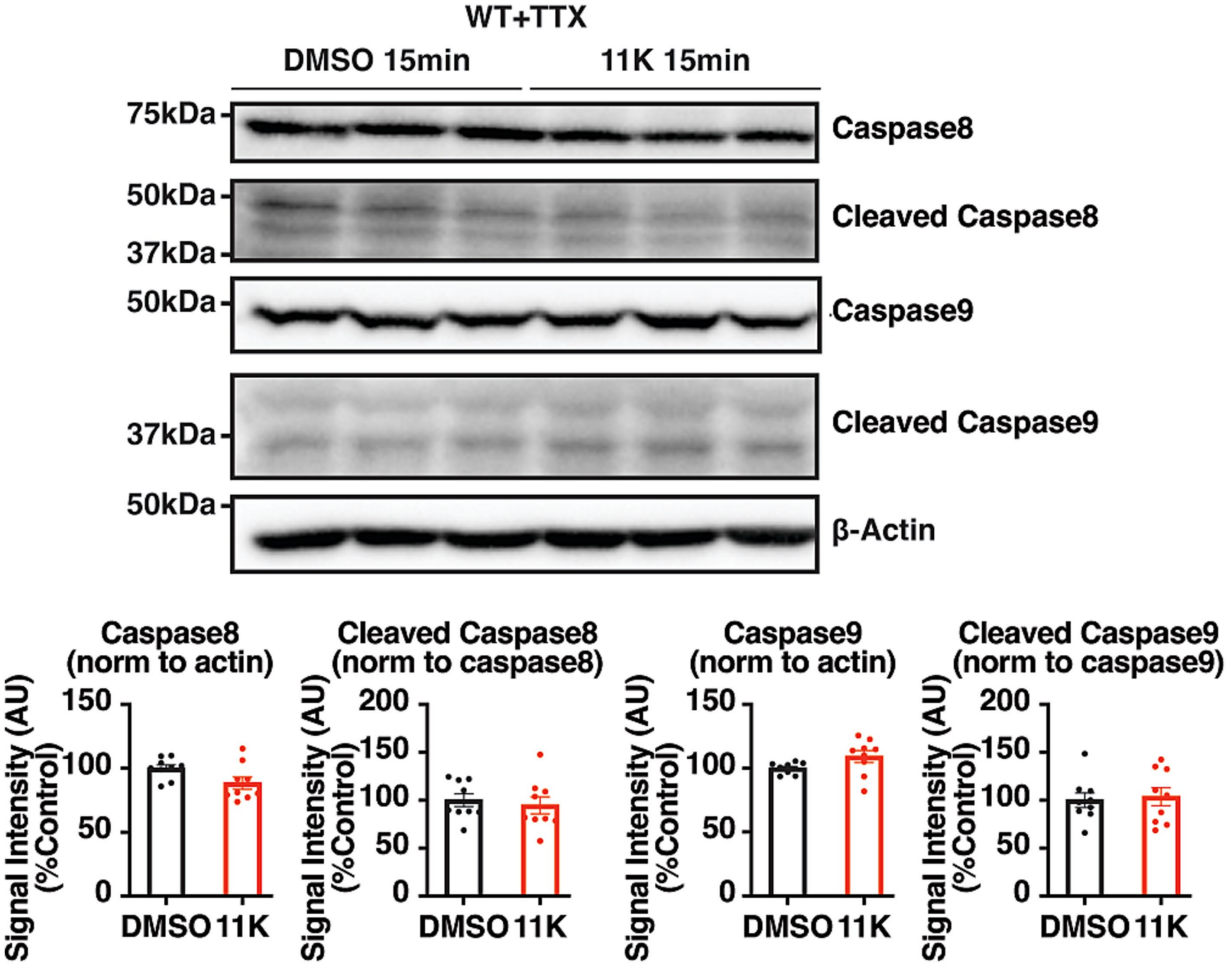
Characterization of the role of neuronal activity in the 11 K-induced activation of extrinsic apoptosis. Representative IB images and quantifications of pro- and cleaved caspase 8 and caspase 9 in 350 μm coronal hippocampal acute brain slices from adult WT mice. Brain slices were exposed to 11 K (10 μM) or DMSO (0.1%) with TTX (500 nM) for 15 min, and lysed with 100 μL RIPA lysis buffer per slice. The results were expressed as mean ± the SEM. All data were subjected to the F-test to compare variances on GraphPad Prism (Version 9.2.0). To assess statistical significance, the unpaired two-sample t-tests were performed, and the Welch’s corrections were applied when necessary. Significance of *p* < 0.05 was represented as *, *p* < 0.01 was represented as **, *p* < 0.001 was represented as ***, and *p* < 0.0001 was represented as ****. All replicates were independent biological replicates. *n* = 9 animals per treatment group, 2–3 slices (merged) per animal.

To investigate the regulatory role of KCC2 activity on neuronal viability further, the role of neuronal activity was examined in acute brain slices from adult WT mice exposed to 11 K with TTX, a sodium channel blocker that ablates neuronal action potential firing. The cleavage of caspases was again quantified using IB and compared to the control group exposed to DMSO and TTX. In the presence of TTX, acute inhibition of KCC2 did not change the levels of procaspase 8 or cleaved caspase 8 within 15 min, indicating no activation of the extrinsic apoptotic pathway. A 15 min 11 K exposure also did not activate the intrinsic pathway, with no change in procaspase 9 or cleaved caspase 9 (Figure 3). Therefore, cessation of neuronal activity prevented the activation of extrinsic apoptosis by the acute inhibition of KCC2. Taken together, the activation of the extrinsic apoptotic pathway by KCC2 inhibition requires neuronal activity.

### The ablation of the KCC2 interactor C1q does not affect KCC2 distribution, KCC2 expression, or baseline caspase activation

**Figure 4:**
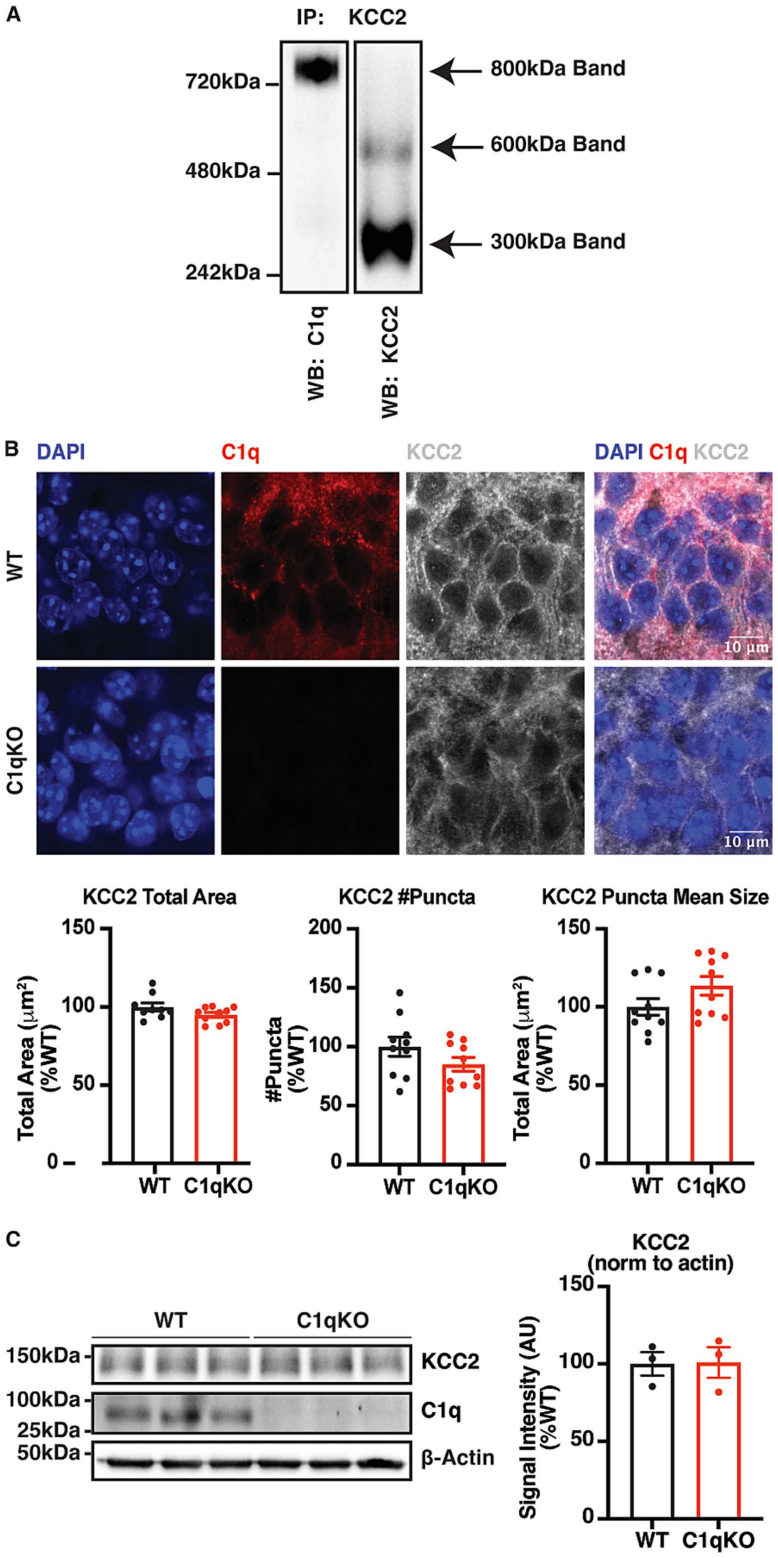
Verification of the KCC2-C1q interaction and characterization of the effect of C1q ablation on KCC2 distribution and expression. **(A)** BN-PAGE images confirming the presence of C1q in the 800 kDa protein complexes of KCC2. KCC2 protein complexes were isolated from forebrain plasma membrane fractions of 8–12-week-old mice by IP, resolved by BN-PAGE, and blotted for C1q. *n* = 1 cohort of 7 animals merged. **(B)** Representative IHC images and quantifications of KCC2 in 30 μm brain slices from adult WT and C1qKO mice. *n* = 3 animals per genotype group, and an average of 10 fields of view per animal, scale bar = 10 μm. The KCC2 total area of one slice was identified as an outlier using the ROUT test and removed from analysis. **(C)** Representative IB images and quantifications of KCC2 and C1q in the forebrain lysate of adult WT and C1qKO mice. Forebrains were extracted, immediately frozen, and lysed with ~2 mL RIPA lysis buffer per hemisphere. *n* = 3 animals per genotype group. The results were expressed as mean ± the SEM. All data were subjected to the F-test to compare variances on GraphPad Prism (Version 9.2.0). To assess statistical significance, the unpaired two-sample t-tests were performed, and the Welch’s corrections were applied when necessary. Significance of *p* < 0.05 was represented as *, *p* < 0.01 was represented as **, *p* < 0.001 was represented as ***, and *p* < 0.0001 was represented as ****. All replicates were independent biological replicates.

C1q is one of the major KCC2 interactors, and copurifies with KCC2 at a KCC2 to C1q ratio of 1: 1.58 (Smalley et al., 2020). C1q and KCC2 interaction was verified by immunoprecipitating KCC2 from brain plasma membrane fractions, followed by BN-PAGE to resolve different molecular species of KCC2. KCC2 migrated as 300 kDa, 600 kDa and 800 kDa bands, representing three distinct species of stable multi-protein complexes. A clear band of C1q appeared in the 800 kDa KCC2 protein complex, confirming the protein–protein interaction of C1q and KCC2 (Figure 4A).

The effect of C1q ablation on KCC2 function was characterized by comparing the distribution patterns and expression levels of KCC2 in the forebrains of adult WT and C1qKO mice using IHC and IB, respectively. IHC confocal images were taken in the hippocampal CA1 region where both KCC2 and C1q are highly expressed. Analysis using the Synapse Counter plugin in FIJI detected no significant difference in KCC2 total area, puncta count, or puncta mean size between genotypes (Figure 4B). Using IB, a clear C1q band was only observed in WT forebrain lysate but not in C1qKO forebrain lysate, verifying the ablation of C1q expression in the KO animals and the high specificity of the C1q antibody used. No significant difference was observed in the expression levels of KCC2 between genotypes (Figure 4C).

**Figure 5:**
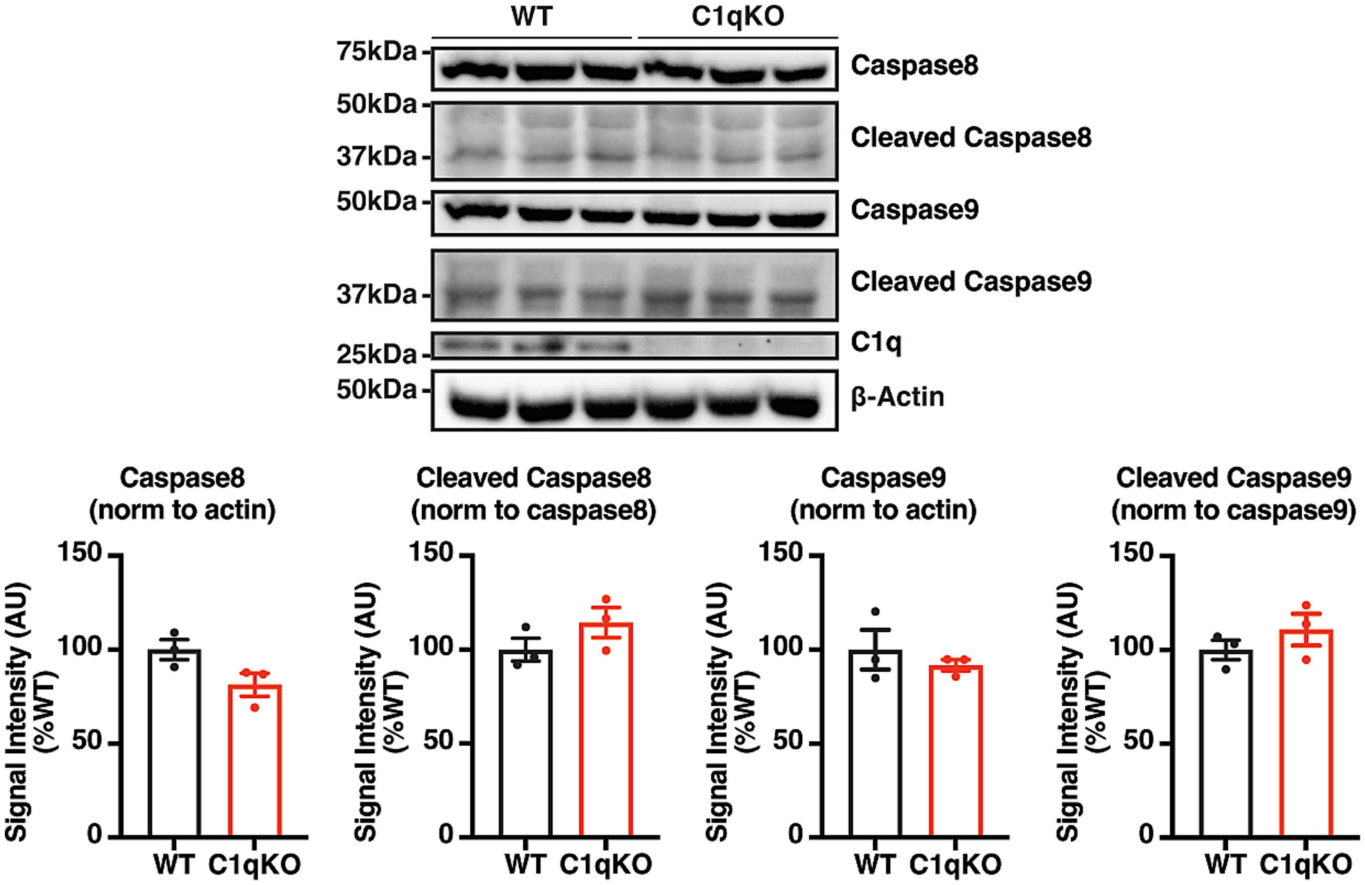
Characterization of the effect of C1q ablation on baseline caspase activation. Representative IB images and quantifications of C1q, caspase 8, and caspase 9 in the forebrain lysate of adult WT and C1qKO mice. Forebrains were extracted, immediately frozen, and lysed with ~2 mL RIPA lysis buffer per hemisphere. n = 3 animals per genotype group. The results were expressed as mean ± the SEM. All data were subjected to the F-test to compare variances on GraphPad Prism (Version 9.2.0). To assess statistical significance, the unpaired two-sample t-tests were performed, and the Welch’s corrections were applied when necessary. Significance of *p* < 0.05 was represented as *, *p* < 0.01 was represented as **, *p* < 0.001 was represented as ***, and *p* < 0.0001 was represented as ****. All replicates were independent biological replicates.

To compare the baseline caspase activity between genotypes, the levels of pro- and cleaved caspases were quantified in the forebrain lysate of adult WT and C1qKO animals. No significant difference was detected in pro- or cleaved caspase levels between genotypes, indicating that the caspase activation at baseline was not significantly different in the presence or absence of C1q (Figure 5). In summary, C1q ablation does not affect KCC2 distribution, KCC2 expression, or baseline caspase activation.

### The activation of the extrinsic apoptotic pathway induced by acute KCC2 inhibition is contingent upon C1q

KCC2 plays a critical role in synaptic inhibition and neuronal viability in adult brains. C1q, a major KCC2 interactor, initiates the classical complement cascade, induces apoptosis via the extrinsic apoptotic pathway, and mediates synaptic pruning in the brain during development and aging (Kaur et al., 2016; Ricklin et al., 2010; Bruce-Keller, 1999; Bhakdi and Tranumjensen, 1987; Aronica et al., 2007; Chu et al., 2010; Itagaki et al., 1994; Laine and Esser, 1989). To characterize the role of C1q in regulating neuronal apoptosis following reduced KCC2 function, acute brain slices from adult C1qKO mice were exposed to 11 K for 15 min. The cleavage of caspases was quantified using IB and normalized to the DMSO control group.

**Figure 6:**
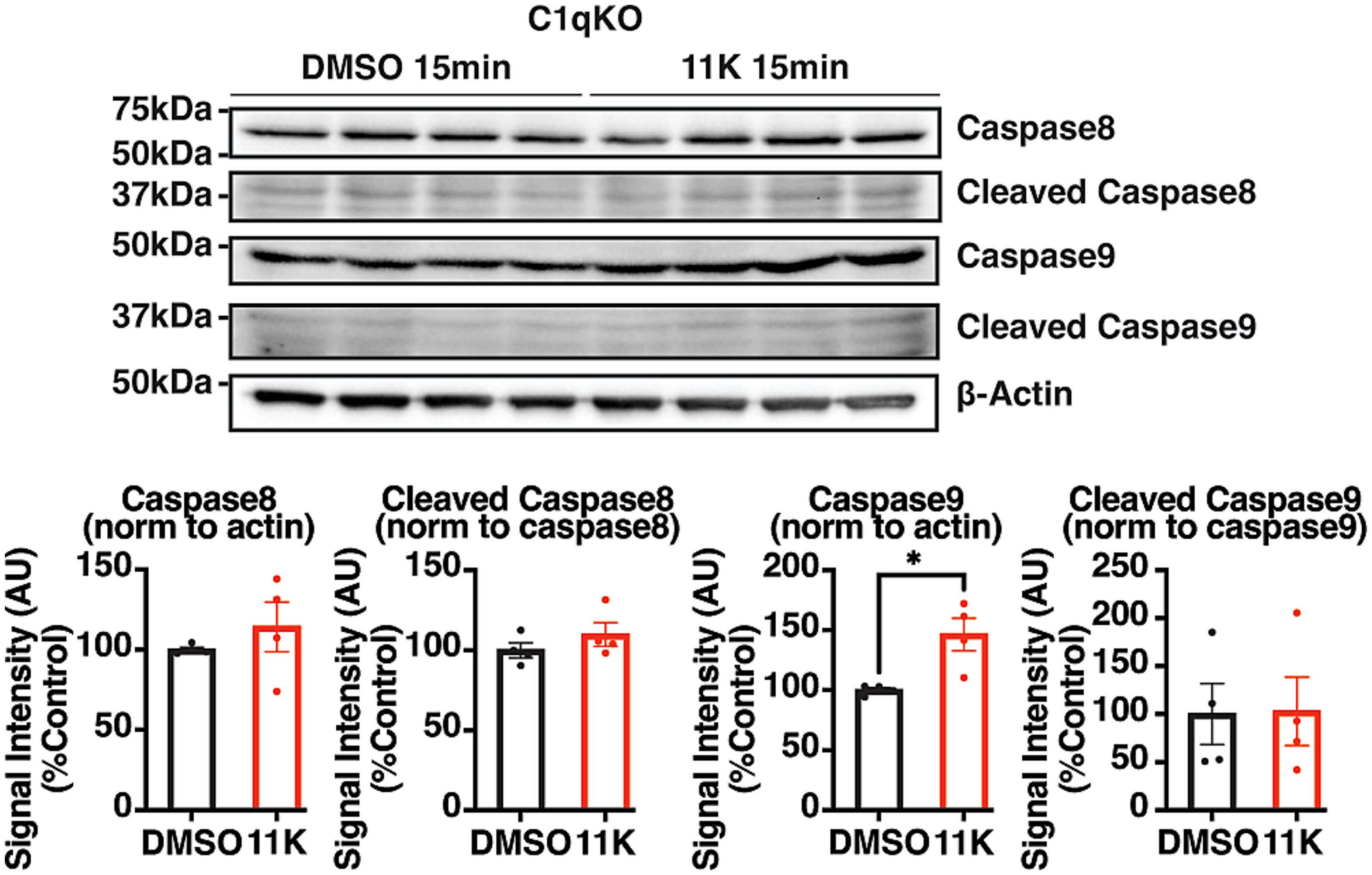
Characterization of the role of C1q in the 11 K-induced activation of extrinsic apoptosis. Representative IB images and quantifications of pro- and cleaved caspase 8 and caspase 9 in 350 μm coronal hippocampal acute brain slices from adult C1qKO mice. Brain slices were exposed to 11 K (10 μM) or DMSO (0.1%) for 15 min, and lysed with 100 μL RIPA lysis buffer per slice. The results were expressed as mean ± the SEM. All data were subjected to the F-test to compare variances on GraphPad Prism (Version 9.2.0). To assess statistical significance, the unpaired two-sample t-tests were performed, and the Welch’s corrections were applied when necessary. Significance of *p* < 0.05 was represented as *, *p* < 0.01 was represented as **, *p* < 0.001 was represented as ***, and *p* < 0.0001 was represented as ****. All replicates were independent biological replicates. *n* = 4 animals per treatment group, 2–3 slices (merged) per animal.

IB analysis detected no significant difference between treatment groups in the levels of either procaspase 8 expression or cleaved caspase 8. Procaspase 9 expression levels were significantly increased within 15 min of 11 K exposure in C1qKO acute brain slices, while cleaved caspase 9 showed no difference between treatment groups (Figure 6). An increase in procaspase 9 expression and no change in cleaved caspase 9 is consistent with no activation of the intrinsic apoptotic pathway. These results suggest that C1q plays a critical role in the activation of extrinsic apoptosis activation.

### Kainic acid and glutamate selectively activate the extrinsic apoptotic pathway

**Figure 7:**
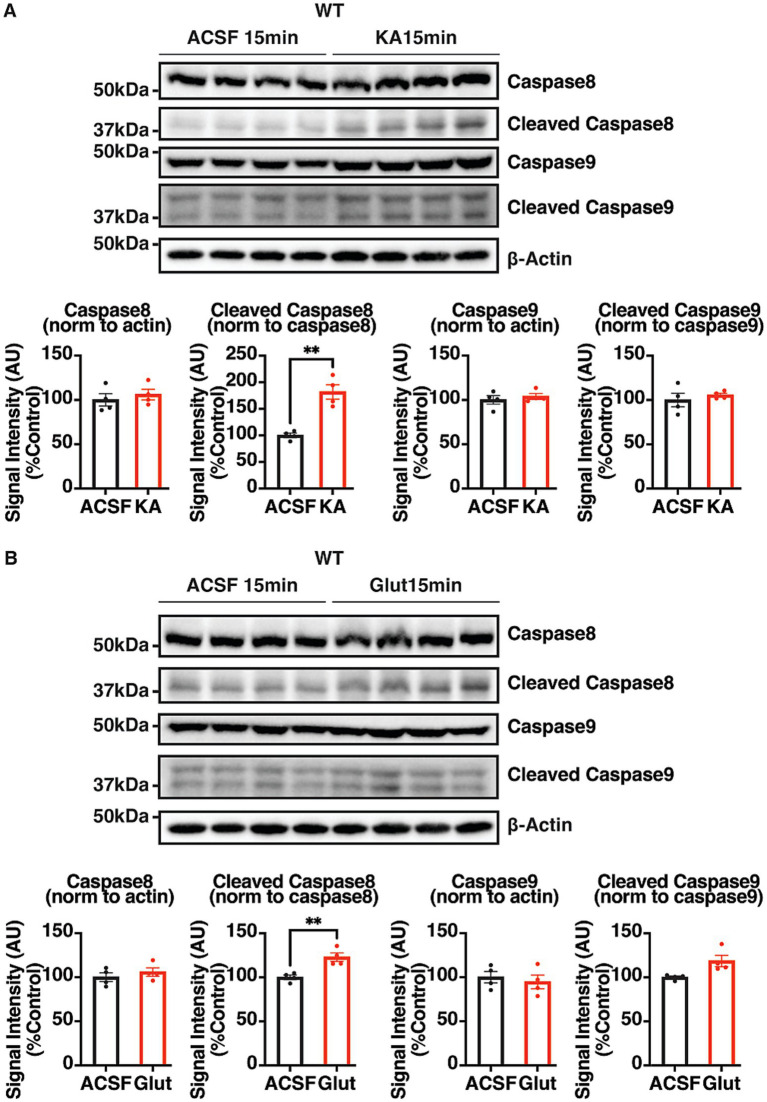
Verification of KA- and glutamate-induced activation of the extrinsic apoptotic pathway. Representative IB images and quantifications of pro- and cleaved caspase 8 and caspase 9 in 350 μm coronal hippocampal acute brain slices from adult WT mice. Brain slices were exposed to **(A)** KA (300 μM) and **(B)** glutamate (300 μM) or aCSF for 15 min, and lysed with 100 μL RIPA lysis buffer per slice. The results were expressed as mean ± the SEM. All data were subjected to the F-test to compare variances on GraphPad Prism (Version 9.2.0). To assess statistical significance, the unpaired two-sample t-tests were performed, and the Welch’s corrections were applied when necessary. Significance of *p* < 0.05 was represented as *, *p* < 0.01 was represented as **, *p* < 0.001 was represented as ***, and *p* < 0.0001 was represented as ****. All replicates were independent biological replicates. *n* = 4 animals per treatment group, 2–3 slices (merged) per animal.

KA, an agonist of glutamate receptors, has been extensively used to induce excitotoxicity in neurons (Magloczky and Freund, 1993; Pollard et al., 1994; Wang et al., 2005), and has been shown to reduce KCC2 expression. To characterize the role of C1q in regulating apoptosis further, 300 μM KA was applied for 15 min to acute brain slices to induce epileptiform activity. To measure KA-induced apoptosis. The cleavage of caspases was quantified using IB and compared to the aCSF control group. KA exposure significantly increased the levels of caspase 8 cleavage within 15 min without affecting the levels of procaspase 8 expression, indicating a rapid activation of the extrinsic apoptotic pathway. No change was observed in the levels of procaspase 9 expression or caspase 9 cleavage (Figure 7A).

The experiment was repeated with 300 μM glutamate treatment for 15 min in WT acute brain slices. Similar to the effect of KA treatment, glutamate significantly increased caspase 8 cleavage with no difference observed in procaspase 8 expression between treatment groups. No change in the levels of procaspase 9 or cleaved caspase 9 were observed (Figure 7B). Therefore, 15 min KA or glutamate exposure in acute slices selectively activates the extrinsic apoptotic pathway.

### The activation of the extrinsic apoptotic pathway induced by KA and glutamate exposure is contingent upon C1q

**Figure 8:**
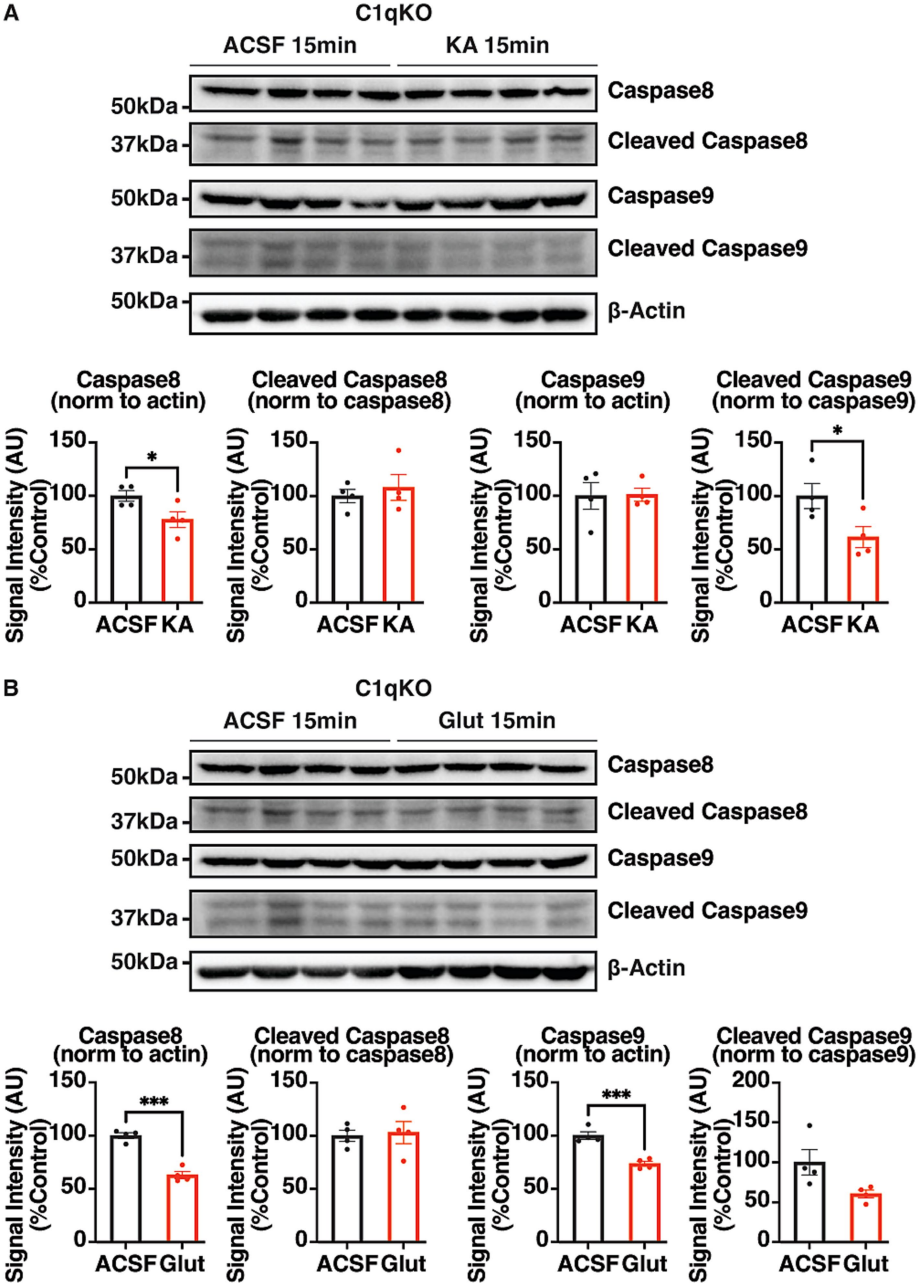
Characterization of the role of C1q in the KA- and glutamate-induced activation of extrinsic apoptosis. Representative IB images and quantifications of pro- and cleaved caspase 8 and caspase 9 in 350 μm coronal hippocampal acute brain slices from adult C1qKO mice. Brain slices were exposed to **(A)** KA (300 μM) and **(B)** glutamate (300 μM) or aCSF for 15 min, and lysed with 100 μL RIPA lysis buffer per slice. The results were expressed as mean ± the SEM. All data were subjected to the F-test to compare variances on GraphPad Prism (Version 9.2.0). To assess statistical significance, the unpaired two-sample *t*-tests were performed, and the Welch’s corrections were applied when necessary. Significance of p < 0.05 was represented as *, *p* < 0.01 was represented as **, *p* < 0.001 was represented as ***, and *p* < 0.0001 was represented as ****. All replicates were independent biological replicates. *n* = 4 animals per treatment group, 2–3 slices (merged) per animal.

To characterize the role of C1q in KA- and glutamate-induced apoptosis, the KA and glutamate treatments were repeated in C1qKO acute brain slices for 15 min, and the levels of pro- and cleaved caspases were compared between treatment groups. In C1qKO brain slices, 15 min of 300 uM KA and glutamate exposure both reduced procaspase 8 expression levels, but no cleavage of caspase 8 was observed, indicating no activation of the extrinsic apoptotic pathway occured (Figure 8). The expression of procaspase 9 was significantly reduced by 15 min of glutamate exposure. Taken together, KA- and glutamate-induced activation of extrinsic apoptosis is contingent upon C1q.

### KCC2 loss and apoptotic cell death in response to *in vivo* KA exposure is dependent on C1q

**Figure 9:**
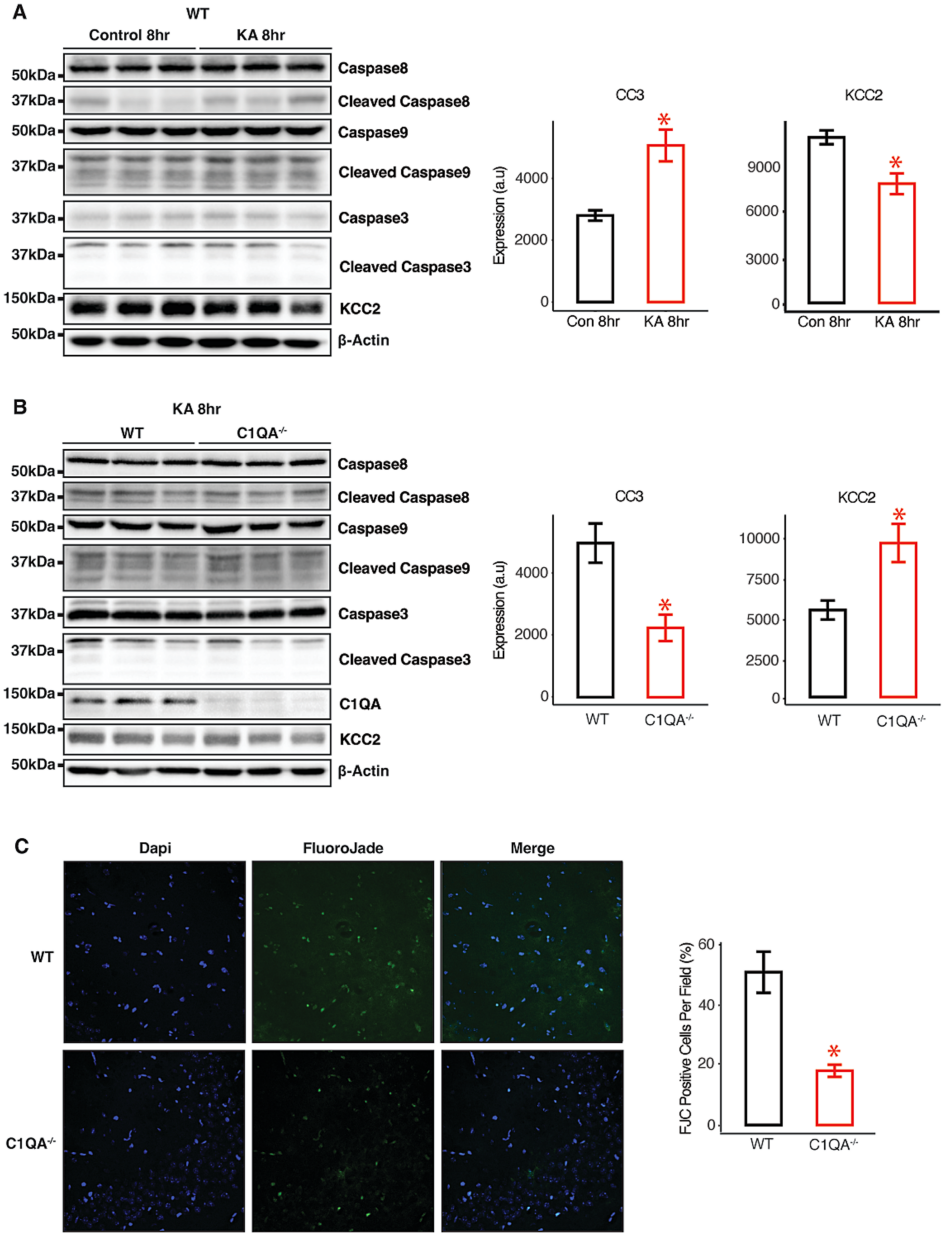
KA-induced apoptosis *in vivo* is dependent on C1q. **(A)** Immunoblots and quantifications of forebrain from WT mice exposed to 20 mg/kg KA for A 2 h or 8 h. **(B)** Immunoblots and quantifications of forebrain from WT or C1qKO mice exposed to 20 mg/kg KA for 8 h B. **(C)** Representative FlouroJade C staining and quantifications from sections of WT and C1qKO mice exposed to 20 mg/kg KA for 8 h. *p < 0.05, *n* = 3.

Finally, we exposed WT mice to KA via intraperitoneal injection. The mice were monitored and sacrificed at either 2 h or 8 h post injection. The forebrain was harvested, and caspase cleavage was measured by IB. No significant changes were observed in the total or cleaved expression of caspase 8 or caspase 9. However, a significant increase in the expression of cleaved caspase 3 was observed in the WT mice at 8 h, accompanied by a significant reduction in the total expression of KCC2 (Figure 9A). The experiment was then repeated in WT and C1qKO mice and forebrain tissue harvested at 8 h post KA injection. C1qKO mice showed significantly lower caspase 3 cleavage compared to WT mice, as well as significantly higher expression of KCC2 (Figure 9B). The same mice were used to prepare brain sections to stain with Fluoro-Jade C (FJC), and image using confocal microscopy (Figure 9C). Cells positive for FJC have been shown to be in the process of apoptotic or necrotic cell death. Significantly fewer FJC positive cells were observed in C1qKO mice following KA exposure. Taken together, these data demonstrate that C1qKO neurons are resistant to KA induced apoptotic cell death and loss of KCC2 expression.

## Discussion

Neuronal hyperexcitability can cause seizures and epilepsy that subsequently result in irreversible neuronal loss. In many cases, seizures can be pharmacologically controlled, but there is a need for improved therapies. KCC2 activators are an emerging therapeutic class that can, in principle, increase KCC2 activity during neuronal hyperactivity, where KCC2 expression is diminished leading to neuronal apoptosis. Our knowledge of the mechanisms by which KCC2 loss can induce apoptosis is therefore of critical importance for the development of biomarkers and further drug development of this novel therapeutic class. Here, for the first time, we show that acute inhibition of KCC2 by the potent and specific KCC2 inhibitor 11 K induces neuronal death in acute brain slices, through selectively activating the extrinsic apoptotic pathway. Here we demonstrated that this process is contingent upon neuronal activity and complement C1q, a major KCC2 interactor. KA- and glutamate-induced excitotoxicity also selectively activates the extrinsic apoptotic pathway in acute brain slices, which we demonstrated is also contingent upon C1q. Although we observed that C1q plays an important role in the KCC2-mediated apoptotic cascade, C1q ablation does not affect KCC2 distribution, KCC2 expression, or caspase activation at baseline. Finally, we demonstrated that 8 h following KA IP injection, reduced KCC2 expression is observed in the mouse forebrain, along with increased caspase 3 cleavage, that is rescued in C1qKO mice. Taken together, this provides a convincing picture of the importance of the C1q/KCC2 complex in mediating neuronal apoptosis in response to hyperexcitability.

C1q-dependent induction of apoptosis has only been directly observed in cancer cell lines, therefore this is the first study to investigate the role of C1q in regulating post-seizure apoptosis in the brain (Hong et al., 2009; Kaur et al., 2016). While the exact mechanism of how the C1q/KCC2 complex mediates apoptosis, we hypothesize that KCC2 may act as a sink for binding C1q on its extracellular domain and following the change of KCC2 surface stability during hyperexcitability, C1q could be released to initiate the extrinsic apoptotic pathway. We hypothesized that C1q binds to the extracellular domain of KCC2 based on two observations. First, macroglia are the major source of C1q in the CNS, whereas KCC2 is mainly expressed in principle neurons; secondly, this and previous studies have shown that loss of KCC2 activates the extrinsic apoptotic pathway, indicating that the signal originates from outside of the cells (Kontou et al., 2021). The models developed here indicate important neuro-immune interactions in seizure-induced neuronal loss. KCC2 appears to be a key regulator of complement-mediated neuronal death via sequestering C1q and preventing the activation of the downstream complement and apoptotic pathway cascades.

It is well established that intrahippocampal ambient glutamate levels increase sharply before and during seizures; this glutamate excitotoxicity during seizures impairs KCC2 function and leads to apoptotic neuronal cell death (Pollard et al., 1994; Ankarcrona et al., 1995; During and Spencer, 1993; Lee et al., 2011; Kitamura et al., 2008). Numerous studies have found that status epilepticus induced by KA reduces KCC2 activity, surface stability, and membrane clustering (Lee et al., 2011; Chamma et al., 2013; Silayeva et al., 2015). The underlying mechanism in the regulation of neuronal activity on KCC2 surface expression is through KCC2 phospho-regulation, which is crucial for modulating its function; dysregulation of KCC2 phosphorylation mediates the development and severity of epileptic seizures (Chamma et al., 2013; Silayeva et al., 2015; Pisella et al., 2019; Friedel et al., 2015; Kahle et al., 2013). KCC2 activity and surface stability are particularly enhanced via direct phosphorylation by Ca^2+^/phospholipid-dependent protein kinase C (PKC) at residue Ser^940^ in the C-terminal intracellular domain (Chamma et al., 2013; Lee et al., 2007). During the glutamate pulse, NMDA receptor activity and Ca^**2+**^ influx cause the loss of KCC2 surface stability via the protein phosphatase 1 (PP1)-dependent dephosphorylation of Ser^940^ (Ankarcrona et al., 1995; Lee et al., 2011). Therefore, 11 K-induced epileptiform activity and KA/glutamate-induced excitotoxicity both reduce KCC2 surface stability, releasing C1q, and extrinsic apoptotic cell death. The mechanism by which hyperexcitability reduces KCC2 surface expression and function is not fully understood, but as KCC2 contains a calpain cleavage site in its c-terminus, it is possible that that c-terminal tail is cleaved following hyperexcitability-induced calpain activation, resulting in KCC2 internalization. Ablation of action potential firing by TTX prevents the epileptiform activity and the reduction of KCC2 surface stability, avoiding the release of C1q and the activation of extrinsic apoptosis. We also observed a difference in the caspase cascades between acute slices and *in vivo* exposure to KA. Caspase 8 cleavage was observed in acute slices, but not *in vivo.* This is likely related to the difference in time post KA exposure that experimental samples were taken. In acute slices samples were assayed 15 min after KA exposure. At this timepoint we capture the transient cleavage of procaspase 8 to cleaved caspase 8. In contrast tissue from mice exposed to KA were harvested and assayed after 2 h or 8 h. By these timepoints, the caspase cascade had progressed past the cleavage of procaspase 8 to cleaved caspase 8 to the terminal stages of apoptosis where procaspase 3 is cleaved to cleaved caspase 3.

Due to the meaningful implications of C1q/KCC2 interaction to neuro-immune pathways regulating seizure-induced neuronal loss and inflammatory injuries to surrounding tissue, further research is necessary to verify and refine the hypothetic KCC2-C1q docking model. For instance, fluorescein isothiocyanate (FITC)-labeled purified C1q could be added to cells expressing KCC2 to investigate the direct binding of C1q to KCC2. Cell-type specific sequencing could be used to identify the cell types involved in the production and release of C1q. Although no abnormal behavior or premature death was observed in the constitutive C1qKO mouse model used in this study, other studies have reported spontaneous atypical absence seizures, autoimmune diseases, increased levels of pro-inflammatory markers in the prefrontal cortex, and increased sensory axon turning within the spinal cord after spinal cord injury (Chu et al., 2010; Trendelenburg et al., 2004; Peterson et al., 2015; Madeshiya et al., 2022). In future studies, *C1qa^flox^* can be used to control C1q expression with higher temporal and regional precision. This conditional knock-out rodent model would enable the examination of C1q’s role in KCC2 mediated neuronal viability at different developmental stages, brain regions, and cell types.

The loss of KCC2 function leads to the lack of therapeutic efficacy of common antiepileptic drugs in the treatment of TLE, and seizure-induced neuronal cell death underlies long-term brain injury (Semah et al., 1998; Stephen et al., 2001). Based on the pivotal role of KCC2 in mediating synaptic inhibition and neuronal survival, KCC2 activation has been shown to prevent benzodiazepine resistant refractory seizures and reduce neuronal cell death after KA-induced seizures (Jarvis et al., 2023). This study demonstrated the pivotal role of complement C1q in KCC2-mediated neuronal survival and helped advance our knowledge of the molecular mechanism of post-seizure neuronal death. Here we only studied the role of the C1q/KCC2 axis on the induction of extrinsic apoptosis in the brain. While we know that C1q is largely expressed by microglia and KCC2 is largely expressed in neurons, significant questions remain regarding the nature of the C1q/KCC2 interaction and how this interaction is modulated during different stages of microglial activation. This will be the focus of future studies. The critical role of KCC2 function and neuro-immune interactions via C1q in regulating apoptosis following seizures will help promote the development of potential immune-based therapeutic applications to alleviate neuronal damage in epilepsy, and aid the production and evaluation of the novel KCC2 activator therapeutic class.

### Experimental procedures

#### Animal care

Animal studies were performed according to protocols approved by the Institutional Animal Care and Use Committee (IACUC) of Tufts Medical Center. 8–12-week-old male and female mice were bred and kept on a 12 h light/dark cycle with *ad libitum* access to food and water at Tufts University School of Medicine’s Division of Laboratory Animal Medicine facility. Mice were housed 1–5 per cage on ventilated racks with gender isolation.

KCC2^fl^ mice (*SlC12A5*lox/lox) have been backcrossed on the C57BL/6 J background for at least 10 generations (Kelley et al., 2018; Melon et al., 2018). *Constitutive C1qa knock-out (C1qKO)* mice lacking exon 3 of the *C1qa* gene, B6(Cg)-*C1qa^tm1d(EUCOMM)Wtsi^*/TennJ were purchased from the Jackson Laboratory. PCR genotyping was carried out in house with an expected product size of 426 bp for wild-type and 543 bp for KCC2^FL^ mice, and 214 bp for wild-type and 302 bp for *C1qKO* mice. The following primers were used for PCR:

KCC2^FL^

5′ – ATG AGT AGC AGA TCC CAT AGG CGA ACC - 3′

5′ – GTA GGT GAC ATC ATT ACC GAG AAC CGT C – 3′ (Melon et al., 2018)

C1qa knock-out

5′ - CCA GAA AGT GCT TAA AGA AAC CA - 3′

5′ - CCT CTC TGA GCC TCT GCT TC - 3′

5′- AGG ACC CTC ATG CTG ATT TG - 3′

#### Antibodies

The following primary antibodies were used for immunoprecipitation (IP), immunoblot/western blot (IB/WB), immunocytochemistry (ICC), or immunohistochemistry (IHC): KCC2 (IP - 1:9.33, IB - 1:1000, IHC- 1:500, mouse, NeuroMab 75–013), Caspase 8 (IB - 1:1000, rabbit, Cell Signaling 4790S), Caspase 9 (IB - 1:1000, mouse, Cell Signaling 9,508 T), *β*-actin (IB – 1:5000 or 1:10000, mouse, Sigma A1978), C1q (IB - 1:1000, IHC - 1:500, rabbit, abcam182451), NeuN (ICC - 1:500, guinea pig, Synaptic Systems 266,004), and GFP (ICC - 1:500, chicken, abcam13970). The following secondary antibodies were used for IB, ICC, or IHC: donkey anti-mouse conjugated HRP (IB - 1:5000, Jackson ImmunoResearch Laboratories), donkey anti-rabbit conjugated HRP (IB - 1:5000, Jackson ImmunoResearch Laboratories), goat anti-guinea pig Alexa Fluor 647 (ICC - 1:1000, Thermo Fisher A-21450), goat anti-chicken Alexa Fluor 488 (ICC - 1:1000, Thermo Fisher A-11039), goat anti-mouse Alexa Fluor 647 (IHC - 1:1000, Thermo Fisher A-21235), and goat anti-rabbit Alexa Fluor 555 (IHC - 1:1000, Thermo Fisher A-21428).

#### Primary neuron culture

Mouse cortical and hippocampal mixed cultures were created from P0 mouse pups as previously described (Kelley et al., 2018; Terunuma et al., 2010). Briefly, P0 mice were anesthetized on ice and the brains removed and dissected in Hank’s buffered salt solution (HBSS) (Thermo Fisher Scientific, Waltham, MA, United States) supplemented with 10 mM HEPES. The cortices and hippocampi were trypsinized and triturated to dissociate the neurons. Viable cells were counted using a hemocytometer and trypan blue staining, followed by plated on poly-l-lysine-coated coverslips at a density of 10^5^ cells/ml in Neurobasal-A media. The final cell counts were around 125 K cells per coverslip/well. All primary cells were kept in the incubator (Thermo Fisher Scientific) at 37°C with 5.0% CO_2_. For KCC2^fl^ cultures, at DIV3, 10^5^ genomic copies per cell of CamKII AAV-GFP or CamKII AAV-Cre (Addgene, Watertown, MA, United States) virus were added to the media. After 24 h, the media was replaced with new conditioned media. At DIV18–21, primary neurons were treated with either dimethyl sulfoxide (DMSO) (0.1%, 12611S Cell Signaling, Beverly, MA, United States) or 11 K (1 μM, VU0463271 [N-cyclopropyl-N-(4-methyl-2-thiazolyl)-2-[(6-phenyl-3-pyridazinyl)thio]acetamide] AstraZeneca, Cambridge, United Kingdom) dissolved in original media for 60 min before the media was replaced with new conditioned media. After 24 h in new conditioned media, cells were washed in 1X phosphate buffered saline (PBS, 10X Solution, Thermo Fisher Scientific) once and fixed in 4% paraformaldehyde (PFA, Electron Microscopy Services, Hatfield, PA, United States) in 1X PBS for 10 min at room temperature. They were then placed in 1X PBS at 4°C until being processed for terminal deoxynucleotidyl transferase dUTP nick end labeling (TUNEL) assay and ICC.

#### Acute brain slices

Murine brains were isolated and sliced into 350 μm coronal hippocampal acute slices in slicing solution (126 mM NaCl, 2.5 mM KCl, 1.25 mM NaH_2_PO_4_, 2 mM MgCl_2_, 0.5 mM CaCl_2_, 1.5 mM Na-pyruvate, 10 mM glucose, 26 mM NaHCO_3_) on ice using a vibratome (VT1000S Leica, Wetzlar, Germany). The brain slices were recovered for 1 h in artificial cerebrospinal fluid (aCSF) (126 mM NaCl, 2.5 mM KCl, 1.25 mM NaH_2_PO_4_, 2 mM MgCl_2_, 0.5 mM CaCl_2_, 1 mM glutamine, 1.5 mM Na-pyruvate, 10 mM glucose, 26 mM NaHCO_3_) at 32°C. Treatment solutions were made with DMSO (0.1%, Cell Signaling), 11 K (10 μM, AstraZeneca), TTX (500 nM, 1,078 Tocris Bioscience, Bristol, United Kingdom), KA (300 μM, 78,050 Cayman Chemical, Ann Arbor, MI, United States), or glutamate (300 μM, G5889 Sigma-Aldrich, St. Louis, MO, United States) in aCSF. Slicing solution, aCSF and treatment solutions were continuously bubbled with carbogen (95% O_2_/5% CO_2_, Airgas, Radnor, PA, United States). Following 15-min of treatment, slices were immediately frozen on dry ice and stored at −80 for IB.

#### Immunoblotting

Sodium dodecyl sulfate polyacrylamide gel electrophoresis (SDS-PAGE) was carried out as previously described (Schiavon et al., 2018). Briefly, Acute brain slices or murine forebrains were homogenized on ice in cold RIPA lysis buffer (50 mM Tris, 150 mM NaCl, 0.1% SDS, 0.5% sodium deoxycholate, and 1% Triton X-100, pH 7.4) supplemented with mini cOmplete protease inhibitor (Roche, Basel, Switzerland) and PhosSTOP phosphatase inhibitor (Roche) tablets. Protein concentration was measured using a Bradford assay (Bio-Rad, Hercules, CA, United States). Samples were diluted to the same concentration in RIPA buffer and Laemmli 2 × Concentrate Sample Buffer (Sigma-Aldrich), and boiled for 3 min at 95°C before 10-50 μg of protein being loaded onto a 7–15% tris-glycine polyacrylamide gel depending on the molecular mass and relative abundance of the target protein. Proteins were then transferred onto nitrocellulose membranes, blocked in 5% milk in tris-buffered saline 0.1% Tween-20 (TBS-T, 20 mM Tris–HCl, 137 mM NaCl, 0.1% Tween-20, pH 7.6) for 1 h, washed with TBS-T, and then probed with primary antibodies prepared in TBS-T overnight (see the antibodies section for dilution information). The membranes were washed and incubated for 1 h at room temperature with HRP-conjugated secondary antibodies (1,5,000 – Jackson ImmunoResearch Laboratories, West Grove, PA, United States). The blots were developed using SuperSignal west dura extended duration substrate (Thermo Fisher Scientific 34,076) or SuperSignal west femto maximum sensitivity substrate (Thermo Fisher Scientific 34,095), and imaged using a ChemiDoc MP (Bio-Rad). Resolved protein bands from raw images were analyzed using Fiji, where band intensity was quantified using the densitometry feature. Where possible, biological replicates were run on the same gels for comparison, and the area under the curve was calculated for each band, normalized to *β*-actin as a loading control, and further normalized to the corresponding control condition. Average signal and standard error of the mean (SEM) were calculated for each treatment group and t-tests were carried out using GraphPad Prism (Version 9.2.0) for statistical comparison of protein expression.

#### Plasma membrane isolation

Plasma membrane isolation was carried out as previously described (Smalley et al., 2020; Suski et al., 2014). Specifically, murine forebrains (from 7 mice) were isolated in 1X PBS and collected in dissection buffer (225 mM mannitol, 75 mM sucrose, 30 mM Tris–HCl, pH 7.4) on ice. Tissues were transferred to homogenization buffer stored on ice containing; 225 mM mannitol, 75 mM sucrose, 0.5% (wt/vol) bovine serum albumin (BSA), 0.5 mM EGTA, 30 mM Tris–HCl, pH 7.4 supplemented with mini cOmplete protease inhibitor (Roche) and PhosSTOP phosphatase inhibitor (Roche) tablets. The brains were homogenized using 14 strokes of a Dounce homogenizer.

All the following centrifugation steps were carried out at 4°C in centrifuge (Avanti J-20 XP Beckman Coulter, Brea, CA, United States). The samples were initially centrifuged at 800 × g for 5 min in a JS 5.3 rotor to facilitate the removal of nuclei and non-lysed cells. After discarding the pellet and filling up to 15 mL with homogenization buffer, the supernatant was spun again at 800 × g for 5 min to remove residual nuclei and non-lysed cells. The supernatant was then centrifuged at 10,000 × g for 10 min in a JA 25.50 rotor to remove mitochondria. The pellet was discarded, and the supernatant was spun again at 10,000 × g for 10 min to remove mitochondrial contamination. The plasma membrane fraction was then pelleted at 25,000 × g for 20 min. Following resuspension in 20 mL of dissection buffer, the plasma membrane fraction was spun again at 25,000 × g for 20 min to remove cytosolic and ER/Golgi contamination.

#### Immunoprecipitation

IP was carried out as previously described (Smalley et al., 2020). Specifically, 150 μL per batch protein G dynabeads (Thermo Fisher Scientific) were washed three times with phosphate buffered saline with 0.05% Tween-20 (PBS-Tween). The beads were resuspended in 250 μL PBS-Tween and incubated overnight at 4°C on the wheel rotator with 30 μL of KCC2 antibody crosslinked to 150 μL of protein G dynabeads, an experimentally predetermined optimal bead: antibody ratio (Smalley et al., 2020). The antibody was crosslinked onto the beads by washing twice with triethanolamine (TEA, 0.2 M, pH 8.2, Sigma-Aldrich), and then incubated for 30 min with dimethyl pimelimidate dihydrochloride (DMP, 40 mM, Sigma-Aldrich) in TEA at room temperature on the rotator. The beads were transferred to Tris (50 mM, pH 7.5, VWR, Radnor, PA, United States) and incubated at room temperature for a further 15 min on the rotator. The beads were washed three times with PBS-Tween and resuspended in 250 μL of resuspension of plasma membrane pellet in 500 μL of Triton lysis buffer (150 mM NaCl, 10 mM Tris, 0.5% Triton X-100, pH 7.5, supplemented with mini cOmplete protease inhibitor (Roche) and PhosSTOP phosphatase inhibitor (Roche) tablets). The IP reaction was incubated overnight at 4°C on the wheel rotator. On the next day, the beads were washed three times with PBS-Tween and eluted with 37.5 μL of soft elution buffer (0.2% (wt/vol) SDS, 0.1% Tween-20, 50 mM Tris–HCl, pH = 8.0) for 30 min at room temperature (Antrobus and Borner, 2011).

#### BN-PAGE

BN-PAGE was carried out as previously described (Smalley et al., 2020). Protein samples were eluted in 37.5 μL soft elution buffer, and diluted in 12.5 μL 4x NativePAGE sample buffer (Thermo Fisher Scientific) and 2 μL 5% G-250 sample additive (Thermo Fisher Scientific), making a total volumn of 52 μL. Samples were loaded onto 4–16% NativePAGE gradient gels (Thermo Fisher Scientific) along with 5 μL of NativeMark unstained protein standard (Thermo Fisher Scientific) and run using a mini gel tank (Thermo Fisher Scientific). Gels were run for 2–3 h in NativePAGE cathode and anode running buffers made from NativePAGE running buffer (20X, Thermo Fisher Scientific) and NativePAGE cathode buffer additive (20X, Thermo Fisher Scientific). For immunoblotting, proteins were transferred to PVDF membranes (Sigma-Aldrich) overnight. The membranes were then fixed in 8% acetic acid, washed with ultrapure water, and air-dried before being briefly destained with 100% methanol. The membranes were then blocked, immunoblotted, and imaged as described above.

#### TUNEL assay and immunocytochemistry

The TUNEL assay is based on the incorporation of modified dUTPs by the enzyme terminal deoxynucleotidyl transferase (TdT) at the 3’-OH ends of fragmented DNA, a terminal hallmark of apoptosis. The TUNEL assay was performed on fixed primary neurons, using Click-iT Plus TUNEL Assay Kits for *In Situ* Apoptosis Detection with Alexa Fluor 594 (Thermo Fisher Scientific). ICC was subsequently carried out according to the kit instruction. Briefly, fixed primary neurons were blocked for 1 h in blocking solution (3% BSA in 1X PBS). Cells were exposed to primary and then fluorophore-conjugated secondary antibodies (Alexa Fluor 488 and 647, Thermo Fisher Scientific) diluted in blocking solution for 1 h each at room temperature in the dark (see antibodies section for dilution information). The coverslips were then washed in blocking solution, dried, and mounted onto microscope slides with ProLong Gold (Thermo Fisher Scientific). The samples were imaged using a Nikon Eclipse Ti (Nikon Instruments, Melville, NY, United States) confocal microscope using a 20 × air objective lens. Image settings were manually assigned for each fluorescent channel. For image processing, the background was subtracted for each fluorescent channel on Fiji Software. TUNEL positive and NeuN positive WT neurons, and TUNEL positive and GFP positive KCC2^flox^ neurons were manually counted per image, averaged for 5 images per coverslip, and normalized to the DMSO treated group from the same preparation.

#### Immunohistochemistry

Whole mouse brains were harvested and drop-fixed in 4% PFA (Electron Microscopy Services) solution overnight. PFA-fixed brains were cryoprotected in 30% sucrose solution prior to OCT embedding. 30 μm coronal brain sections were obtained from a Leica CM1900 cryostat, and underwent 5 min antigen retrieval in sodium citrate buffer (10 mM, Sigma-Aldrich) at 95°C. Fixed brain sections were washed and blocked for 2 h in Fab blocking solution (1X PBS with 0.5% Triton X-100, 1% Fab fragment goat anti-mouse IgG, 3% (wt/v) BSA, 10% normal goat serum, and 0.2 M glycine). at room temperature. Sections were exposed to primary antibodies diluted in blocking solution (1X PBS with 0.5% Triton X-100, 3% (wt/vol) BSA, 10% normal goat serum, and 0.2 M glycine) at 4°C overnight, and then fluorophore-conjugated secondary antibodies (Alexa Fluor 555 and 647, Thermo Fisher Scientific) diluted in blocking solution for 2 h at room temperature in the dark (see antibodies section for dilution information). The sections were then washed in 1X PBS, and mounted onto microscope slides with ProLong Gold Antifade Mountant with DAPI (Thermo Fisher Scientific). Images of the CA1 hippocampal region were acquired by a Nikon Eclipse Ti (Nikon Instruments) confocal microscope using a 60 × oil immersion objective lens. Image settings were manually assigned for each fluorescent channel. For image processing, puncta counts and colocalization studies were carried out as previously described (Kontou et al., 2021). Briefly, the puncta count, puncta mean size, total area of KCC2 were quantified using the Synapse Counter plugin in FIJI (Version 2.1.0), averaged for 3 images per slice, 2–4 slices per animal, and normalized to WT animals (Dzyubenko et al., 2016; Schindelin et al., 2012). 1,024 ×1,024 confocal images were auto-thresholded using the Otsu Thresholding method. The rolling ball radius (background subtraction) and maximum filter parameters were set to 7 and 1, respectively. Default colocalization settings were used that accept 33–100% overlap between C1q and KCC2 puncta. Average and SEM were calculated for each genotype and t-tests carried out using GraphPad Prism (Version 9.2.0) for statistical comparison.

#### Statistics and reproducibility

In many of the experiments we have normalized to the control group while maintaining the variance in the control group for correct statistical analysis downstream. For immunocytochemistry this is achieved by precise plating of cell numbers and then normalizing the cell number of the control group around the mean to 100%. The experimental group can then be expressed as a percentage of the control group. For western blots this is achieved by running all the samples to be compared on the same gel. The expression of each protein in the control group can then be normalized around the mean to 100% and the experimental group can then be expressed as a percentage of the control group. The results are expressed as mean ± the SEM. All data were subjected to the F-test to compare variances on GraphPad Prism (Version 9.2.0). To assess statistical significance, the unpaired two-sample t-test was performed, and the Welch’s correction was applied when necessary. The ROUT test was applied to identify any outlier which was removed from analysis. Significance of *p* < 0.05 is represented as *, *p* < 0.01 is represented as **, *p* < 0.001 is represented as ***, and *p* < 0.0001 is represented as ****. All replicates are independent biological replicates. For immunocytochemistry experiments, *n* = 4 individual primary cultures from 6 to 12 pups per genotype. For immunoblot experiments, *n* = 3–9 was used, where samples were derived either from individual murine forebrains, or from acute brain slices treatments with 2–3 slices (merged) per animal. For immunoprecipitation experiment, *n* = 1 cohort of 7 animals merged. For immunohistochemistry experiment, *n* = 3 was used, where samples were derived from individual murine whole brains.

## Data Availability

The original contributions presented in the study are included in the article/supplementary material, further inquiries can be directed to the corresponding author/s.

## Glossary

aCSF: Artificial cerebrospinal fluid
BN-PAGE: Blue-native polyacrylamide gel electrophoresis
BSA: Bovine serum albumin
CNS: Central nervous system
DIV: Days *in vitro*
DMP: Dimethyl pimelimidate dihydrochloride
DMSO: Dimethyl sulfoxide
FITC: Fluorescein isothiocyanate
GABA_A_Rs: type A *γ*-aminobutyric acid receptors
HBSS: Hank’s buffered salt solution
HEK: Human embryonic kidney
IACUC: Institutional Animal Care and Use Committee
IB: Immunoblot
ICC: Immunocytochemistry
IHC: Immunohistochemistry
IP: Immunoprecipitation
KA: Kainic acid
KCC2: Potassium chloride co-transporter 2
LC–MS/MS: Liquid chromatography–tandem mass spectrometry
PARP: Poly (ADP-ribose) polymerase
PBS: Phosphate buffered saline
PBS-Tween: Phosphate buffered saline with 0.05% Tween-20
PFA: Paraformaldehyde
PKC: Protein kinase C
PP1: Protein phosphatase 1
SDS-PAGE: Sodium dodecyl sulfate polyacrylamide gel electrophoresis
SEM: Standard error of the mean
TBS-T: Tris-buffered saline 0.1% Tween-20
TdT: Terminal deoxynucleotidyl transferase
TEA: Triethanolamine
TTX: Tetrodotoxin
TUNEL: Terminal deoxynucleotidyl transferase dUTP nick end labeling
